# Recurrent pain among young and early midlife employees – the role of workload and health-related factors

**DOI:** 10.1186/s13690-025-01595-3

**Published:** 2025-04-17

**Authors:** Pi Fagerlund, Jukka-Pekka Jousilahti, Anne Kouvonen, Katri Salo, Jatta Salmela, Tea Lallukka

**Affiliations:** 1 Department of Public Health, University of Helsinki, Helsinki, Finland; 2 Faculty of Social Sciences, University of Helsinki, Helsinki, Finland

**Keywords:** Pain, Health behaviors, Employees, Workload

## Abstract

**Background:**

Recurrent pain is associated with subsequent sickness absence and disability retirement, however, its modifiable risk factors have been less frequently studied among young and early midlife employees. This study aimed to identify workload and health-related factors associated with recurrent pain among young and early midlife employees, as this could help specify targets for pain prevention in this age group to mitigate its adverse consequences.

**Methods:**

The Helsinki Health Study data, covering 3245 (79% women) Finnish municipal employees who were 19–39-years-old at phase 1, were collected by surveys in 2017 (phase 1) and 2022 (phase 2). Data on workload and health-related factors were derived from phase 1, and data on pain from both surveys. The associations of workload and health-related factors with pain recurrence were investigated using multinomial logistic regression analysis.

**Results:**

Recurrent pain was reported by 25% of the employees. Pain either only at phase 1 or at phase 2 was reported by 16% and 18% of the participants, respectively, whereas 41% of the participants reported no pain at either phase. High physical and mental workload, non-daily vegetable consumption, obesity, and average sleep duration other than 7–8 h were associated with recurrent pain.

**Conclusions:**

Recurrent pain affects a substantial proportion of young and early midlife employees. Improving physical and psychosocial working conditions, through workplace interventions and multilevel interventions to support healthy behaviors, are recommended to prevent recurrent pain.

**Supplementary Information:**

The online version contains supplementary material available at 10.1186/s13690-025-01595-3.

**Table:** 

Text box 1. Contributions to the literature	
• Recurrent pain has been little studied among younger employees although pain is associated with work disability and stronger associations have been observed for recurrent pain. • Over a 5-year period, one fourth of younger Finnish municipal employees reported recurrent pain and multiple modifiable health related factors and workload were associated with recurrent pain. • Supporting healthy lifestyle choices and ensuring appropriate workload at workplaces could be means to reduce recurrent pain among younger employees.	

## Background

Pain conditions are common in working populations and contribute to sickness absence [24], disability [23], productivity loss, and healthcare expenditures [13]. To experience pain occasionally is normal, however, recurrent or chronic pain may become disabling and confer an increased risk of sickness absence, even among young and early midlife adults [4, 16]. The prevalence of chronic pain is higher in older age groups, but it also affects younger individuals, and pain earlier in adulthood has been associated with pain that persists into later life [3, 10]. A previous Finnish study showed that over 40% of under 40-year-old employees reported current pain, and 20% had had chronic pain for more than 3 months [3]. Additionally, reporting pain at a single occasion was associated with subsequent sickness absence in this age group [4]. However, stronger associations with work disability have been found for recurrent pain [15, 19]. Thus, preventing pain, as well as identifying modifiable factors that may increase the risk of recurrent pain already among younger employees, could be a means to support their current and future work ability. Recurrent pain and factors associated with it are generally less frequently studied among younger employees [16]. Identifying modifiable predictors of recurrent pain is crucial for the secondary prevention of pain, as well as for enhancing our knowledge of possible intervention targets that can help prevent recurrent pain and thus promote work ability.

Longitudinal studies on factors associated with trajectories of pain have primarily been conducted in cohorts of midlife or older employees or mixed ages [1, 20, 25]. The literature on work- and health-related risk factors for recurrent pain among younger employees, who are expected to remain in work for many more decades, is scarce. However, the importance of preventing pain early has been indicated by a Norwegian study showing that the number of pain sites remained relatively stable over a 12-year follow-up, including among the younger individuals [10]. Younger employees may have different underlying causes and risk factors of their pain, as compared to older employees, therefore, a separate focus on young and early midlife employees is justified.

We aimed to investigate pain recurrence during a 5-year follow-up among young and early midlife employees, and to identify workload and health-related factors that are associated with pain recurrence, as this could enable the identification of targets of primary and secondary prevention of persistent pain among employees in this age group.

## Methods

### Data collection and study population

**Fig. 1:**
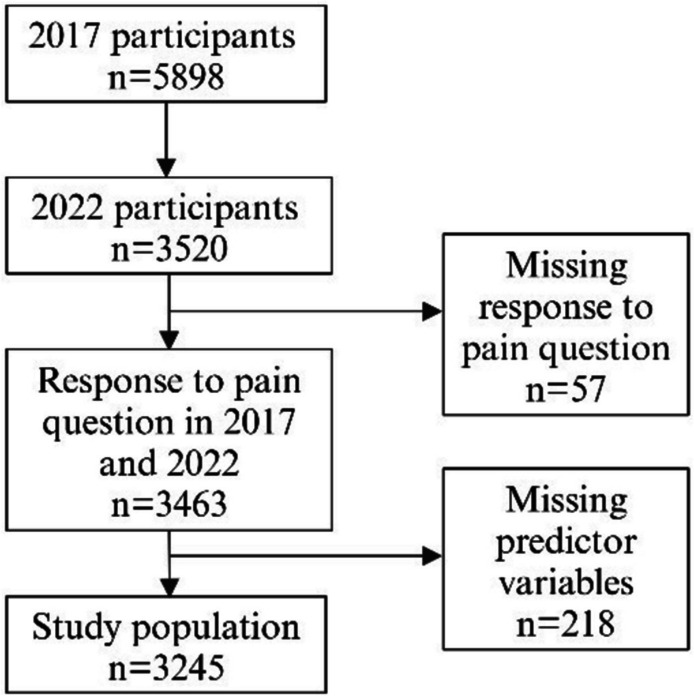
Flowchart diagram of the selection of the study population. Created with *BioRender.com*

The data were collected as a part of the Helsinki Healthy Study and cover employees of the City of Helsinki, Finland, who were 19–39 years old at baseline in 2017 (phase 1) and had been employed by the City of Helsinki for at least 4 months with a contract of at least 50% of full-time employment [15, 19]. In phase 1, responses were obtained by a web-based questionnaire (58%), mail (29%), and telephone interviews (13%), the latter of which were conducted if the respondent had not answered despite reminders. Of the initial target population (*N* = 11,459), a total of 5898 participants responded (response rate 51%) to the phase 1 survey [19]. The target population for the 2022 follow-up (phase 2) were those who had responded at phase 1 (*n* = 3520, response rate 60%). The questionnaire at phase 2 was similar to that in phase 1, and responses were obtained by web-based questionnaire (61%), mail (30%), and telephone interviews (9%). The final study population of 3245 participants was obtained by excluding those who had missing answers to pain questions in either phase (*n* = 57) or missing information on workload or health-related factors (*n* = 218) (Fig. 1).

### Variables

Variables used in this study include pain status at phases 1 and 2, as well as socio-demographic, work-, and health-related variables at phase 1. The variables used for the analyses were restricted to those enquired about in both the online and mailed questionnaires and in the phone interview, which included a smaller set of questions. This was due to phone respondents more often being manual workers, and excluding this group would risk introducing selection bias and underrepresentation of this group [15, 19].

#### Pain

In both survey phases, employees were asked “Are you suffering from any pains or aches right now” (yes/no). A four-class variable was created based on the responses “No pain”, “Pain only at phase 1”, “Pain only at phase 2”, and “Recurrent pain”, meaning pain at both phase 1 and 2.

#### Workload

Perceived physical and mental workload were enquired by the question “What is your job like [physically/mentally]?”, and response alternatives were given on a four-level scale ranging from very light to very strenuous. Physical workload was classified into a three-level categorical variable so that the iii) “quite strenuous” and iv) “very strenuous” categories were merged. Mental workload was also classified into a three-level categorical variable so that the categories i) “very light” and ii) “fairly light” were merged. Different cut-off points for heavy physical and mental workload were used due to a high proportion of the participants reporting a very strenuous mental workload. A similar classification of these items has been used previously [14].

#### Health-related factors

The included health-related factors were smoking, alcohol consumption, vegetable consumption, body mass index (BMI) and average sleep duration. The frequency of smoking was enquired by the question “Do you smoke cigarettes?”. The response alternatives ranged from “daily” to “never smoked”. A dichotomized variable was created to separate those who smoked regularly or occasionally (“smokers”) from respondents who had stopped smoking or had never smoked (“non-smokers”). Alcohol consumption was assessed with the question “Which of the following options best describes your current beer, wine and spirits consumption?” Responses were given on a 10-point scale ranging from “I don’t drink alcohol” to “daily or almost daily”. Responses were further classified into two categories: “less than weekly” and “at least weekly”. Vegetable consumption was enquired by the question “How often do you consume the following food items [fresh vegetables, root vegetables, and fresh salad]? Think about the past four weeks”. Seven response alternatives were provided, ranging from “not in the past 4 weeks” to “2 times or more a day”. The frequency of consumption was dichotomized into “at least daily” and “less than daily”. BMI was calculated based on respondents’ self-reported height and weight and dichotomized into < 30 (no obesity) and ≥ 30 kg/m^2^ (obesity) [27]. The average sleep duration was enquired by the question “How much on average do you sleep within a 24-h period?” and responses were given as the number of hours and minutes. The average sleep duration was classified into three categories: “7–8 h”, “ < 7 h” and “ > 8 h”.

#### Background variables

Background variables were age, gender, marital status, and education level reported at phase 1. Age was calculated based on the year of birth and classified as “19–29 years” and “30–39 years”. Gender was reported as “woman” and “man”. Marital status was dichotomized as “married or cohabiting” and “single, divorced, or widowed”. Missing observations (*n* = 11) were merged with the reference group “married or cohabiting” to not exaggerate the effect and not lose individuals due to a lack of response to a covariate. Education level was reported on a six-level scale ranging from basic education to licentiate or doctoral degree and classified as “low” (equivalent to upper secondary or vocational school) and “high” (Bachelor’s degree or higher). Missing observations (*n* = 9) were merged with the “low” education group since missingness is more common among individuals in lower socioeconomic positions.

### Statistical methods

Background variables, workload, and health-related factors were cross-tabulated by gender for descriptive purposes (Table 1). Background variables, workload, and health-related factors were cross-tabulated by pain status, and the associations were analyzed by the Pearson Chi-squared test with *p*-values (Table 2). No systematic gender interaction effect with workload and health-related factors on pain recurrence was observed, and the statistical power did not suffice for gender-stratified analyses, which is why the associations by gender are not shown separately in the main analyses. However, workload and health-related factors were cross-tabulated by change of pain status separately for both genders and presented in Supplementary Table 1. The included background and predictor variables showed, in general, low multicollinearity (variance influence factor mean 1.11) and low correlation as measured by Spearman correlation coefficients, the highest being 0.34 between physical workload and education level (Supplementary Table 2). The associations of workload and health-related factors with pain recurrence between phases 1 and 2 were analyzed using multinomial logistic regression analysis. In Model 1, the analyses were adjusted for age and gender. The analyses in Model 2 were further adjusted for education level and marital status, as well as mutually adjusted for physical and mental workload, smoking, alcohol consumption, vegetable consumption, obesity, and average sleep duration. Statistical analyses were conducted using StataMP 18 statistical software (StataCorp LLC, College Station, TX, USA).

**Table 1:** Characteristics of the Helsinki Health Study population (*n* = 3245). Sociodemographic factors, workload, health-related factors and pain status by gender

	**Men**	**Women**	**All**	***p*-value**
	n ***(%)***	n ***(%)***	n ***(%)***	
**Gender**	695 (21)	2550 (79)	3245	
**Age**				0.078
19–29 years	176 (25)	732 (29)	908 (28)	
30–39 years	519 (75)	1818 (71)	2337 (72)	
**Marital** status				0.059
Married or cohabiting	491 (71)	1705 (67)	2196 (68)	
Unmarried, other	204 (29)	845 (33)	1049 (32)	
**Education level**				< 0.001
High	404 (58)	1836 (72)	2240 (69)	
Low	291 (42)	714 (28)	1005 (31)	
**Physical workload**				< 0.001
Light	175 (25)	536 (21)	711 (22)	
Medium	347 (50)	1116 (44)	1463 (45)	
Heavy	173 (25)	898 (35)	1071 (33)	
**Mental workload**				< 0.001
Light	224 (32)	514 (20)	738 (23)	
Medium	369 (53)	1579 (62)	1948 (60)	
Heavy	102 (15)	457 (18)	559 (17)	
**Smoking**				0.173
Non-smoker	530 (76)	2006 (79)	2536 (78)	
Smoking	165 (24)	544 (21)	709 (22)	
**Alcohol consumption**				< 0.001
< weekly	393 (57)	1909 (75)	2302 (71)	
≥ weekly	302 (43)	641 (25)	943 (29)	
**Vegetable consumption**				< 0.001
≥ daily	385 (55)	1903 (75)	2288 (71)	
< daily	310 (45)	647 (25)	957 (29)	
**Obesity**				0.906
No	591 (85)	2173 (85)	2764 (85)	
Yes	104 (15)	377 (15)	481 (15)	
**Average sleeping hours**				< 0.001
7–8 h	486 (70)	1794 (70)	2280 (70)	
< 7 h	171 (25)	489 (19)	660 (20)	
> 8 h	38 (5)	267 (10)	305 (9)	
**Pain**				< 0.001
No pain	333 (48)	1007 (39)	1340 (41)	
Pain only at phase 1 (2017)	110 (16)	462 (18)	572 (18)	
Pain only at phase 2 (2022)	116 (17)	418 (16)	534 (16)	
Recurrent pain	136 (20)	663 (26)	799 (25)

**Table 2:** Characteristics of the Helsinki Health Study participants by pain status at phases 1 (2017) and 2 (2022) (*n* = 3245)

	**No pain**	**Pain only at phase 1**	**Pain only at phase 2**	**Recurrent pain *(%)***	***p*-value**
	n *(%)*	n *(%)*	n *(%)*	n *(%)*	
**Total**	1340 (41)	572 (18)	534 (16)	799 (25)	
**Gender**					< 0.001
Woman	1007 (39)	462 (18)	418 (16)	663 (26)	
Man	333 (48)	110 (16)	116 (17)	136 (20)	
**Age**					0.473
19–29 years	389 (43)	163 (18)	149 (16)	207 (23)	
30–40 years	951 (41)	409 (18)	385 (16)	592 (25)	
**Marital status**					0.377
Married or cohabiting	922 (42)	389 (18)	364 (17)	521 (24)	
Unmarried, other	418 (40)	183 (17)	170 (16)	278 (27)	
**Education level**					< 0.001
High	1001 (45)	386 (17)	363 (16)	490 (22)	
Low	339 (34)	186 (19)	171 (17)	309 (31)	
**Physical workload**					< 0.001
Light	342 (48)	123 (17)	112 (16)	134 (19)	
Medium	619 (42)	258 (18)	245 (17)	341 (23)	
Heavy	379 (35)	191 (18)	177 (17)	324 (30)	
**Mental workload**					< 0.001
Light	347 (47)	109 (15)	140 (19)	142 (19)	
Medium	816 (42)	361 (19)	311 (16)	460 (24)	
Heavy	177 (32)	102 (18)	83 (15)	197 (35)	
**Smoking**					0.010
Non-smoker	1082 (43)	443 (17)	415 (16)	596 (24)	
Smoker	258 (36)	129 (18)	119 (17)	203 (29)	
**Alcohol consumption**					0.146
< weekly	929 (40)	407 (18)	375 (16)	591 (26)	
≥ weekly	411 (44)	165 (18)	159 (17)	208 (22)	
**Vegetable consumption**					0.002
≥ daily	974 (43)	399 (17)	391 (17)	524 (23)	
< daily	366 (38)	173 (18)	143 (15)	275 (29)	
**Obesity**					< 0.001
No	1,192 (43)	484 (18)	460 (17)	628 (23)	
Yes	148 (31)	88 (18)	74 (15)	171 (36)	
**Average sleep**					< 0.001
7–8 h	1027 (45)	397 (17)	371 (16)	485 (21)	
< 7 h	210 (32)	120 (18)	106 (16)	224 (34)	
> 8 h	103 (34)	55 (18)	57 (19)	90 (30)	

### Ethical considerations

The study protocol of the Helsinki Health Study has obtained a positive statement from the ethics committee of the Faculty of Medicine, University of Helsinki, and has been approved by the City of Helsinki. The respondents have, by participation in the Helsinki Health Study, agreed to the questionnaire data being used for scientific research purposes, and the data is being handled in line with the data protection statement of the Helsinki Health Study [8]. As part of the Helsinki Health Study, this observational study did not require a separate ethical approval, however, ethical aspects of the study have been considered in line with the Declaration of Helsinki.

## Results

### Descriptive results

Of the study population of 3245 employees, women accounted for almost four-fifths, and approximately three-fourths of the participants were aged 30–39 years at phase 1 (Table 1). Over two-thirds had a higher education degree, which women were more likely than men to have attained. Women reported higher physical and mental workloads than men. Weekly or more frequent alcohol consumption was more frequent among men, who also consumed vegetables less frequently. A smaller proportion of women reported an average sleep duration of < 7 h, and it was more common for women to sleep > 8 h, as compared to men. The prevalences of smoking and obesity did not differ statistically significantly between genders. Women were overrepresented in the groups of employees reporting pain.

For women, associations between all studied indicators of workload and health-related factors, except alcohol consumption, with recurrent pain were identified (Supplementary Table 1). For men, statistically significant associations were only observed for mental workload and average sleep duration.

Employees who had no pain at both phases constituted 41% of the study population, whereas 25% reported recurrent pain (Table 2). Employees with pain only at phase 1 comprised 18% of the participants and employees with pain only at phase 2 comprised 16% of the participants. Women and employees with a lower education level were more likely to report recurrent pain and less likely to report no pain at both phases 1 and 2. Pain status did not differ statistically significantly by age group and marital status. All considered work- and health-related factors, except from alcohol consumption, differed by pain status, so that those with lower physical and mental workloads, without obesity, non-smokers, those with daily vegetable consumption, or average daily sleep of 7–8 h were more likely to report no pain at both phases 1 and 2. Conversely, employees who at phase 1 reported high mental or physical workload, smoking, less than daily vegetable consumption, obesity, or average daily sleep less than 7 or more than 8 h were more likely to report recurrent pain.

### Associations of workload and health-related factors with pain

The results from multinomial regression analyses showed that both workload and health-related factors were associated with recurrent pain (Table 3). Heavy physical workload (Odds ratio [OR] 1.38, 95% confidence interval [CI] 1.05─1.81) and medium (OR 1.36 95%, CI 1.06─1.75) or heavy mental workload (OR 1.78, 95% CI 1.28─2.46) at phase 1 were associated with reporting pain only at phase 1, as compared to employees with no pain at both phases, after adjusting for age and gender. After further adjustment for sociodemographic factors, health-related factors, and workload, the association of physical workload with pain was eliminated but remained for both medium and heavy mental workload. Among the health-related factors, having obesity (OR 1.47, 95% CI 1.11─1.96) and average sleep duration of < 7 h (OR 1.51, 95% CI 1.17─1.94) were associated with reporting pain only at phase 1, when adjusting for age and gender. The associations were slightly attenuated after full adjustments for sociodemographic factors, workload, and other health-related factors.

**Table 3:** Associations of workload and health-related factors with recurrent pain among the Helsinki Health Study participants (*n* = 3245). Results from the multinomial logistic regression analyses; odds ratios (OR) and their 95% confidence intervals (CI) are shown. “No pain” at both phases 1 (2017) and 2 (2022) was used as a reference group

	**Model 1 **^**a**^			**Model 2 **^**b**^		
	**Pain only at phase 1**	**Pain only at phase 2**	**Recurrent pain (phases 1 and 2)**	**Pain only at phase 1**	**Pain only at phase 2**	**Recurrent pain (phases 1 and 2)**
	OR (95% CI)			OR (95% CI)		
**Physical workload**						
Light	1	1	1	1	1	1
Medium	1.16 (0.90─1.49)	1.21 (0.93─1.56)	1.42 (1.12─1.80)	1.04 (0.81─1.35)	1.15 (0.89─1.50)	1.19 (0.93─1.53)
Heavy	1.38 (1.05─1.81)	1.40 (1.06─1.86)	2.22 (1.73─2.86)	1.08 (0.81─1.46)	1.23 (0.91─1.67)	1.49 (1.13─1.96)
**Mental workload**						
Light	1	1	1	1	1	1
Medium	1.36 (1.06─1.75)	0.93 (0.73─1.17)	1.32 (1.05─1.65)	1.40 (1.09─1.81)	0.92 (0.73─1.18)	1.35 (1.06─1.70)
Heavy	1.78 (1.28─2.46)	1.14 (0.82─1.58)	2.62 (1.97─3.48)	1.81 (1.29─2.52)	1.10 (0.79─1.54)	2.57 (1.91─3.46)
**Smoking**						
Non-smoker	1	1	1	1	1	1
Smoker	1.23 (0.97─1.57)	1.21 (0.94─1.54)	1.46 (1.19─1.81)	1.11 (0.86─1.42)	1.12 (0.87─1.44)	1.18 (0.95─1.48)
**Alcohol consumption**						
< weekly	1	1	1	1	1	1
≥ weekly	0.96 (0.77─1.20)	0.99 (0.79─1.24)	0.84 (0.69─1.02)	1.03 (0.82─1.29)	1.07 (0.85─1.34)	0.98 (0.80─1.21)
**Vegetable consumption**						
≥ daily	1	1	1	1	1	1
< daily	1.23 (0.99─1.53)	1.00 (0.79─1.26)	1.56 (1.28─1.89)	1.15 (0.92─1.44)	0.93 (0.73─1.17)	1.35 (1.10─1.66)
**Obesity**						
No	1	1	1	1	1	1
Yes	1.47 (1.11─1.96)	1.30 (0.97─1.76)	2.19 (1.72─2.79)	1.34 (1.00─1.79)	1.22 (0.90─1.65)	1.82 (1.42─2.34)
**Average sleep**						
7–8 h	1	1	1	1	1	1
< 7 h	1.51 (1.17─1.94)	1.42 (1.09─1.84)	2.31 (1.86─2.87)	1.36 (1.05─1.76)	1.34 (1.03─1.75)	1.87 (1.49─2.34)
> 8 h	1.34 (0.95─1.90)	1.50 (1.06─2.12)	1.82 (1.34─2.47)	1.33 (0.94─1.90)	1.50 (1.06─2.13)	1.77 (1.30─2.42)

^a^Model 1: age (continuous) + gender adjusted

^b^Model 2: age (continuous) + gender + education + marital status + smoking + alcohol + vegetables + obesity + sleep + mental workload + physical workload adjusted

After adjusting for age and gender, heavy physical workload (OR 1.40, 95% CI 1.06─1.86) was associated with reporting pain only at phase 2, when compared to employees with no pain. When further adjusting for sociodemographic and health-related factors and mental workload, this association was eliminated. Among the health-related factors, having an average sleep duration of < 7 h (OR 1.42, 95% CI 1.09─1.84) or > 8 h (OR 1.50, 95% CI 1.06─2.12) were associated with reporting pain only at phase 2 after age and gender were adjusted for. The associations remained similar after full adjustments for sociodemographic factors, workload, and other health-related factors.

After adjusting for age and gender, medium physical workload (OR 1.42, 95% CI 1.12─1.80) and heavy physical workload (OR 2.22, 95% CI 1.73─2.86), as well as medium mental workload (OR 1.32, 95% CI 1.05─1.65) and heavy mental workload (OR 2.62, 95% CI 1.97─3.48), were associated with having recurrent pain, as compared to those who reported no pain. In the fully and mutually adjusted Model 2, the association of a medium-level physical workload with recurrent pain was eliminated but remained for heavy physical and mental workload, and medium mental workload. Among the health-related factors, smoking, consuming vegetables less than daily, obesity, and average sleep duration of < 7 h or > 8 h were associated with having recurrent pain after adjusting for age and gender. In Model 2, full and mutual adjustments eliminated the association between smoking and recurrent pain and attenuated the associations for the other health-related factors.

## Discussion

### Main findings

In the present study conducted among young and early midlife municipal employees, recurrent pain was common and reported by 25% of the employees. Pain only at phase 1 was reported by 18%, while 16% reported pain only at phase 2, whereas 41% reported no pain at both phases. Women were more likely to report recurrent pain than men. Of the investigated workload and health-related factors, high physical and mental workload, non-daily vegetable consumption, obesity, and an average sleep duration < 7 h or > 8 h were associated with recurrent pain. Medium and heavy physical workload, obesity and average sleep duration < 7 h were associated with pain only at phase 1, whereas average sleep duration < 7 h or > 8 h were associated with pain only at phase 2.

### Previous studies

There are both public health and financial reasons to investigate risk factors for the onset of pain that persists or recurs and develops into chronic pain, which has consistent associations with sickness absence, disability retirement and early work exit overall across working populations [4, 16, 17, 24]. However, pain may change over time, and thus, measuring pain repeatedly with a longer follow-up and not at only one time point could provide new information about group-level risk factors for recurrent pain, which has also been associated with more sickness absence in previous studies, as compared to pain reported at a single measurement point [15, 19]. Sickness absence poses a financial burden on employers and society and has increased in recent years among younger Finnish municipal employees [11]. Intervening on modifiable factors for recurrent pain could be a means to support work ability among young and early midlife municipal employees who are expected to have decades left in paid employment. However, previous studies focusing on the determinants of recurrent pain are sparse and conducted among heterogeneous study populations. These earlier studies have focused on adolescents’ pain [7] and specific occupations such as food industry workers [20]. There have been no previous studies, to the best of our knowledge, that have focused on young and midlife employees representing a wide spectrum of different occupations.

There are studies indicating that the number of pain sites on a group level varies relatively little over time. Kamaleri et al. [10] studied change in the 12-month prevalence of multisite pain in a Norwegian population-based cohort with a 14-year follow-up [10]. They found that the number of pain sites remained relatively stable, including among the younger individuals. Only 5.4% of the individuals reporting pain at baseline reported no pain 14 years later, indicating that pain tended to persist. In our study, the measure was a point prevalence of pain, and a somewhat lower proportion of the respondents reported pain at phase 2 as compared to phase 1. This may have been caused by selection of healthier employees to phase 2 through a loss of participants due to work disability or ending of the employment. However, a majority of the employees who reported pain at phase 1 had recurrent pain, which aligns with findings by Kamaleri et al. [10] that indicated that reported pain status remained relatively stable over the follow-up. A quarter of the young and early midlife employees reported recurrent pain in this study. A previous cross-sectional study, based on the same cohort as in this study, found a 19%-point prevalence of chronic pain at phase 1 [3]. Additional ad hoc analyses showed that 65% of young and early midlife employees who reported chronic pain (lasting ≥ 3 months) at phase 1 and responded at phase 2 had recurrent pain after 5 years. This indicates that employees with baseline chronic pain are likely to still experience pain after multiple years, but also that chronic pain conditions are dynamic, and remission is possible.

Both workload and health-related factors were associated with recurrent pain among initially 19–39-year-old employees in our study. Similar factors, such as heavy physical and mental workload, low leisure-time physical activity, and overweight at baseline have been associated with a trajectory of higher number of pain sites during a 28-year follow up among Finnish employees initially 44–58 years of age [21]. Hanvold et al. [7] studied technical school students entering working life and found that low socioeconomic position and manual work were associated with a higher number of pain sites among women. Smoking was also associated with a higher number of pain sites, but only among men. In contrast with the study by Hanvold et al. that included electricians, hairdressers and media and design students, our cohort included a high proportion of healthcare workers, such as nurses, practical nurses and childcare workers, who are exposed to both high physical and psychosocial demands at work. In the current cohort dominated by women, both heavy physical and mental workloads were associated with recurrent pain, after adjusting for education level and other sociodemographic, workload, and health-related factors. Thus, work adjustments to improve physical and cognitive ergonomics for employees working with pain could be a means to prevent recurrent pain.

Pain is a typical symptom of musculoskeletal disorders, the prevalence and disease burden of which are expected to globally increase among young and early midlife adults over the coming decades, highlighting the importance of preventive efforts [6]. In our study, we found associations between health-related factors such as obesity, smoking, average sleep duration other than 7–8 h and non-daily vegetable consumption. A study on the disease burden of musculoskeletal disorders among under 39-year-olds found obesity to be a key contributing factor to low back pain [6]. However, the same study showed that the contribution of smoking to low back pain is decreasing over time. In our study, smoking was associated with recurrent pain after adjusting for age and gender, but not in the fully adjusted model. Average sleep duration more or less than 7–8 h was, in the current study, associated with recurrent pain, but the measure used considered only the self-reported average duration of sleep and not sleep quality. However, the finding is in line with the comprehensive body of literature showing an association between sleep-related problems and pain, both of which separately and concomitantly are associated with sickness absence and disability retirement [18, 26]. This study found that non-daily vegetable consumption was associated with recurrent pain, and the association remained after controlling for multiple lifestyle-related factors, such as BMI and education level. This has to our best knowledge not been shown before, although some smaller intervention studies have shown pain improvement by introducing a plant-based diet [2, 9, 12]. The potential positive effects of dietary interventions to manage chronic pain has been shown earlier, however, no specific interventions have been identified as superior [5]. This study was observational, and the finding should be interpreted with caution since unobserved confounding by, for example, occupation differences in dietary habits and pain could be present. More studies exploring this association are welcome.

### Methodological considerations

This study was based on a large cohort of young and early midlife employees working in the municipal sector and representing a broad range of occupational titles of different education levels and employment sectors, with occupational titles ranging from, for example, practical nurses to teachers and physicians. However, a majority worked in the healthcare, education, and social sectors. We also included the phone respondents who, as compared to the online and mail respondents, were more often manual workers. This increased the representativeness of the sample. Not excluding manual workers through selection bias was important due to pain being more prevalent among employees who do manual work [15, 19]. Nonetheless, since phone interviews had to be kept relatively short, they could only include key variables of interest. Therefore, our set of variables available did not include, for example, physical activity or some other potentially relevant variables. Obesity is, however, an overall key determinant of health, including pain [6, 22, 27]. Although we adjusted the analyses for a range of covariates, unmeasured and residual confounding is always a possibility in an observational study and cannot be ruled out. We tested for gender interactions and found no statistically significant differences. Still, women dominate the sample, as 79% of the participants were women, which corresponds well to the gender distribution of the municipal sector in Finland at the time of the surveys. However, the results of the study may not be directly generalized to the male-dominated private sector. In addition, highly educated employees were more likely to respond to the phase 2 survey, meaning that employees with lower education levels, who report more pain, may have been lost during the follow-up.

Pain is a subjective experience and therefore suitable for self-report. The pain measure was based on longitudinal data from two measurement points, distinguishing individuals who have pain that recurs or persists over time from individuals who did not have pain on both occasions and those who experienced pain at either measurement point. Since there was a five-year interval between the surveys, it needs to be acknowledged that pain may have changed or fluctuated between the surveys, and this is missed. However, since we focus on group level, the results are likely to reflect key potentially modifiable work- and health-related factors that are associated with recurrent pain.

### Conclusions

A substantial proportion of young and early midlife Finnish municipal employees experience recurrent pain, which is notable since pain is also a consistent indicator of an elevated risk of work disability. Physical and mental workload, as well as multiple health-related factors, were associated with recurrent pain. Improvement of physical and mental working conditions and multilevel interventions to support a healthy diet, weight management, and good sleep could be a means to reduce the risk of recurrent pain. Future studies could consider occupation differences and use multiple measuring points to provide an even more detailed understanding by identifying trajectories of pain and factors associated with those trajectories.

## Supplementary Information

Supplementary Material 1.

## Data Availability

The Helsinki Health Study survey data cannot be made publicly available due to strict data protection laws and regulations. The data can only be used for scientific research. More information on the survey data can be requested from the Helsinki Health Study research group (kttl-hhs@helsinki.fi).

## Abbreviations

BMI: Body mass index
CI: Confidence interval
OR: Odds ratio
VIF: Variance influence factor
