# Supplementary material for: Recurrent pain among young and early midlife employees – the role of workload and health-related factors
Source: Arch Public Health. 2025 Apr 17;83:108. doi: 10.1186/s13690-025-01595-3 (PMC12004546; doi:10.1186/s13690-025-01595-3)
Supplement: Supplementary file 1 — Supplementary Material 1. [file 13690_2025_1595_MOESM1_ESM.docx]

| **Supplementary table 1.** Gender-specific distributions of workload and health-related factors by pain status. *n<10, not shown. | | | | | |
| --- | --- | --- | --- | --- | --- |
|  | **No pain** | **Pain only at Phase 1** | **Pain only at Phase 2** | **Recurrent pain (Phases 1 and 2)** |  |
|  | n *(%)* | n *(%)* | n *(%)* | n *(%)* | p-value |
| **Women (n=2550)** | 1007 | 462 | 418 | 663 |  |
| **Physical workload** |  |  |  |  | < 0.001 |
| Light | 244 (46) | 96 (18) | 86 (16) | 110 (21) |  |
| Medium | 458 (41) | 205 (18) | 184 (16) | 269 (24) |  |
| Heavy | 305 (34) | 161 (18) | 148 (16) | 284 (32) |  |
| **Mental workload** |  |  |  |  | < 0.001 |
| Light | 235 (46) | 77 (15) | 99 (19) | 103 (20) |  |
| Medium | 636 (40) | 299 (19) | 248 (16) | 396 (25) |  |
| Heavy | 136 (30) | 86 (19) | 71 (16) | 164 (36) |  |
| **Smoking** |  |  |  |  | 0.012 |
| Non-smoker | 822 (41) | 361 (18) | 326 (16) | 497 (25) |  |
| Smoker | 185 (34) | 101 (19) | 92 (17) | 166 (31) |  |
| **Alcohol consumption** | |  |  |  | 0.828 |
| < weekly | 747 (39) | 344 (18) | 313 (16) | 505 (26) |  |
| ≥ weekly | 260 (41) | 118 (18) | 105 (16) | 158 (25) |  |
| **Vegetable consumption** | |  |  |  | 0.002 |
| ≥ daily | 775 (41) | 344 (18) | 325 (17) | 459 (24) |  |
| < daily | 232 (36) | 118 (18) | 93 (14) | 204 (32) |  |
| **Obesity** |  |  |  |  | < 0.001 |
| No | 901 (41) | 392 (18) | 361 (17) | 519 (24) |  |
| Yes | 106 (28) | 70 (19) | 57 (15) | 144 (38) |  |
| **Average sleep** |  |  |  |  | < 0.001 |
| 7–8 h | 781 (44) | 320 (18) | 286 (16) | 407 (23) |  |
| < 7 h | 147 (30) | 90 (18) | 80 (16) | 172 (35) |  |
| > 8 h | 79 (30) | 52 (19) | 52 (19) | 84 (31) |  |
|  |  |  |  |  |  |
| **Men (n=695)** | 333 (48) | 110 (16) | 116 (17) | 136 (20) |  |
| **Physical workload** |  |  |  |  | 0.186 |
| Light | 98 (56) | 27 (15) | 26 (15) | 24 (14) |  |
| Medium | 161 (46) | 53 (15) | 61 (18) | 72 (21) |  |
| Heavy | 74 (43) | 30 (17) | 29 (17) | 40 (23) |  |
| **Mental workload** |  |  |  |  | 0.030 |
| Light | 112 (50) | 32 (14) | 41 (18) | 39 (17) |  |
| Medium | 180 (49) | 62 (17) | 63 (17) | 64 (17) |  |
| Heavy | 41 (40) | 16 (16) | 12 (12) | 33 (32) |  |
| **Smoking** |  |  |  |  | 0.636 |
| Non-smoker | 260 (49) | 82 (15) | 89 (17) | 99 (19) |  |
| Smoker | 73 (44) | 28 (17) | 27 (16) | 37 (22) |  |
| **Alcohol consumption** | |  |  |  | 0.329 |
| < weekly | 182 (46) | 63 (16) | 62 (16) | 86 (22) |  |
| ≥ weekly | 151 (50) | 47 (16) | 54 (18) | 50 (17) |  |
| **Vegetable consumption** | |  |  |  | 0.067 |
| ≥ daily | 199 (52) | 55 (14) | 66 (17) | 65 (17) |  |
| < daily | 134 (43) | 55 (18) | 50 (16) | 71 (23) |  |
| **Obesity** |  |  |  |  | 0.243 |
| No | 291 (49) | 92 (16) | 99 (17) | 109 (18) |  |
| Yes | 42 (40) | 18 (17) | 17 (16) | 27 (26) |  |
| **Average sleep** |  |  |  |  | 0.001 |
| 7–8 h | 246 (51) | 77 (16) | 85 (17) | 78 (16) |  |
| < 7 h | 63 (37) | 30 (18) | 26 (15) | 52 (30) |  |
| > 8 h | 24 (63) | * | * | * |  |

| **Supplementary table 2.** Spearman correlation coefficients and variance influence factors (VIF) | | | | | | | | | | | | |
| --- | --- | --- | --- | --- | --- | --- | --- | --- | --- | --- | --- | --- |
|  | **Age (continuous)** | **Gender** | **Education level** | **Marital status** | **Smoking** | **Alcohol** | **Vegetables** | **Obesity** | **Average sleep** | **Physical workload** | **Mental workload** | **VIF** |
| **Age (continuous)** | 1 |  |  |  |  |  |  |  |  |  |  | 1.10 |
| **Gender** | -0.03 | 1 |  |  |  |  |  |  |  |  |  | 1.10 |
| **Educational level** | 0.14 | 0.12 | 1 |  |  |  |  |  |  |  |  | 1.29 |
| **Marital status** | 0.14 | -0.03 | 0.08 | 1 |  |  |  |  |  |  |  | 1.06 |
| **Smoking** | -0.05 | -0.03 | -0.18 | -0.12 | 1 |  |  |  |  |  |  | 1.09 |
| **Alcohol** | 0.08 | -0.17 | 0.11 | 0.05 | 0.09 | 1 |  |  |  |  |  | 1.07 |
| **Vegetables** | -0.07 | -0.17 | -0.17 | -0.08 | 0.08 | -0.01 | 1 |  |  |  |  | 1.08 |
| **Obesity** | 0.04 | 0.00 | -0.14 | -0.04 | 0.07 | -0.07 | 0.08 | 1 |  |  |  | 1.08 |
| **Average sleep** | -0.03 | 0.03 | -0.07 | -0.06 | 0.03 | -0.08 | 0.06 | 0.06 | 1 |  |  | 1.10 |
| **Physical workload** | -0.14 | 0.08 | -0.34 | -0.04 | 0.07 | -0.15 | 0.05 | 0.04 | 0.06 | 1 |  | 1.03 |
| **Mental workload** | -0.02 | 0.1 | 0.08 | -0.02 | 0.03 | 0.00 | -0.04 | 0.01 | 0.05 | 0.15 | 1 | 1.21 |
|  |  |  |  |  |  |  |  |  |  |  |  | Mean VIF 1.11 |
